# Epidemiologic characteristics of diabetes in children aged 0–14 years in Busan and Gyeonnam Province, Korea (2001–2010)

**DOI:** 10.1186/1687-9856-2013-S1-P13

**Published:** 2013-10-03

**Authors:** Suyoung Hong, Hyunji Kim, Junghyun Lee, Jaeho Yoo

**Affiliations:** 1 Department of Pediatrics, Maryknoll Hospital, Hong Kong; 2 Department of Pediatrics, Wallace Memorial Baptist Hospital, Busan, Korea; 3 Department of Pediatrics, Kosin University Gospel Hospital, Busan, Korea; 4 College of Medicine, Dong-A University, Busan, Korea

## Objective

We performed this study to investigate changes in the incidence of type 1 diabetes mellitus (T1DM) and type 2 diabetes mellitus (T2DM) in children aged below 15 years in Busan and Gyeonnam province, Korea during the period from 2001 to 2010.

## Methods

We sent questionnaires via the post to 4 tertiary and 43 general hospitals in Busan and Gyeonnam province. We received answered questionnaires based on medical records from all tertiary and 11 general hospitals. Three hundred forty four diabetic patients (239 T1DM, 89 T2DM, 16 unclassified DM) who were newly diagnosed from 2001 to 2010 were enrolled. The incidence rates were calculated as the number of cases per 100,000 person-years. The denominator for incidence rate was secondary data from the population registry (Korea National Statistical Office). Ninety five percent confidence intervals (95% CI) was calculated using Poisson distribution. The incidence rate was fitted and test for the linear trend was done by Poisson linear regression model. We also used the Poisson regressions to assess the rate ratio for development of diabetes with year, sex, age group, geographic location. The data were analyzed using SAS 9.2.

## Results

The average crude incidence was 2.01(95% CI: 1.77-2.28) and 0.76(95% CI: 0.61-0.93) for T1DM and T2DM, respectively. There was a significant increasing trend in the incidence of T1DM during the period of 2001 and 2010, with an annual 1.08-fold increase of the rate ratio (95% CI: 1.03-1.12). The trend in the incidence of T2DM increased with an annual 1.23-fold increase of the rate ratio (95% CI: 1.14-1.33). The incidence of T2DM among those aged 10-14 years was rapidly increased during the period of 2001 to 2010 and was higher than that of T1DM in 2010.

The rate ratio for development of T1DM was 1.16-fold higher in males than females and that for those aged 10-14 years was a 2.04-fold higher when compared to those aged 0-4 years. Also, there was an 1.43 increased rate ratio in Busan (urban) compared to Gyeonnam province (suburban). The rate ratio for development of T2DM was 1.29-fold higher in males than females and that for those aged 10-14 years was a 9.59-fold higher when compared to those aged 5-9 years. Also, there was an 1.58 increased rate ratio in Busan (urban) compared to Gyeonnam province(suburban).

## Conclusions

The incidence of T1DM and T2DM have shown a significant increasing trend for last 10 years in Busan and Gyeonnam province, Korea. Our study shows the needs for careful monitoring of incidence and its related risk factors of T2DM in adolescents.

